# Spectrum-Effect Relationships Between Chemical Fingerprints and Antibacterial Effects of Lonicerae Japonicae Flos and Lonicerae Flos Base on UPLC and Microcalorimetry

**DOI:** 10.3389/fphar.2016.00012

**Published:** 2016-02-02

**Authors:** Zhilong Shi, Zhenjie Liu, Chunsheng Liu, Mingquan Wu, Haibin Su, Xiao Ma, Yimei Zang, Jiabo Wang, Yanling Zhao, Xiaohe Xiao

**Affiliations:** ^1^Pharmacy College, Chengdu University of Traditional Chinese MedicineChengdu, China; ^2^China Military Institute of Chinese Medicine, 302 Hospital of People's Liberation ArmyBeijing, China; ^3^School of Chinese Pharmacy, Beijing University of Chinese MedicineBeijing, China; ^4^Liver Failure Therapy and Research Center, 302 Hospital of People's Liberation ArmyBeijing, China

**Keywords:** Lonicerae Japonicae Flos, antibacterial, microcalorimetry, spectrum-effect relationships, UPLC

## Abstract

The traditional Chinese medicines *Lonicerae Japonicae Flos* (LJF, Jinyinhua in Chinese) and Lonicerae Flos (LF, Shanyinhua in Chinese) refer to the flower buds of five plants belonging to the Caprifoliaceae family. Until 2000, all of these were officially listed as a single item, LJF (Jinyinhua), in the Chinese Pharmacopoeia. However, there have recently been many academic controversies concerning the separation and combination of LJF and LF in administrative regulation. Till now there has been little work completed evaluating the relationships between biological activity and chemical properties among these drugs. Microcalorimetry and UPLC were used along with principal component analysis (PCA), hierarchical cluster analysis (HCA), and canonical correlation analysis (CCA) to investigate the relationships between the chemical ingredients and the antibacterial effects of LJF and LF. Using multivariate statistical analysis, LJF and LF could be initially separated according to their chemical fingerprints, and the antibacterial effects of the two herbal drugs were divided into two clusters. This result supports the disaggregation of LJF and LF by the Pharmacopoeia Committee. However, the sample of *Lonicera fulvotomentosa* Hsu et S. C. Cheng turned out to be an intermediate species, with similar antibacterial efficacy as LJF. The results of CCA indicated that chlorogenic acid and 3,4-Dicaffeoylquinic acid were the major components generating antibacterial effects. Furthermore, 3,4-Dicaffeoylquinic acid could be used as a new marker ingredient for quality control of LJF and LF.

## Introduction

Lonicerae Japonicae Flos (LJF, *Lonicera japonica* Thunb., Jinyinhua in Chinese) is one of the most commonly used traditional Chinese medicines. Use of LJF was first recorded in Shen-Nong's Herbals, one of the world's earliest pharmacopeias, and it has been widely used throughout China for disease prevention and treatment. Specifically, LJF has a variety of bioactive effects, including antibacterial, anti-inflammatory (Tae et al., 2003), and antiviral properties as well as liver protection activity (Jiang et al., 2014). LJF is officially listed in the 2015 version of the Chinese pharmacopeia, where it is listed simultaneously with Lonicerae Flos (LF, Shanyinhua in Chinese). They have the same descriptions of flavor, meridian tropism, functions, dosage, and indications in the pharmacopeia (Commission, 2015). LF involves the flowers or buds of four plants: *Lonicera macranthoides* Hand.-Mazz., *Lonicera hypoglauca* Miq., *Lonicera confusa* DC., and *Lonicera fulvotnetosa* Hsu et S. C. Cheng. Few studies of LF exist, and most of them focus only on *L. macranthoides* Hand.-Mazz. (Li et al., 2015). Before the 2000 version of the Chinese pharmacopoeia, both LJF and LF were listed under the same category of LJF (Jinyinhua in Chinese). However, for medical safety, especially in regards to drug injection, the Pharmacopoeia Commission separated these into two herbal drugs. Based on the divergence of chemical ingredients, only *Lonicera japonica* Thunb. is listed in LJF (Jinyinhua in Chinese) in the 2015 version of the Chinese pharmacopoeia, while the other plant sources are listed in LF (Shanyinhua in Chinese). However, due to the similarity of these two drugs in clinical applications, there are many disputes about the administrative regulation of LJF and LF. It is therefore urgent to do further research to determine the pharmaceutical activity of the two herbal drugs.

Sensitive biological methods are needed to explore the diversity and bioactivity of these two medicines. Traditional bioassays are time-consuming and also consume large amounts of materials, and more importantly, they are not able to easily and clearly identify the different bioactivities of closely related species. Microcalorimetry is an ideal method to overcome these challenges because it is a real-time and quantitative means of measuring thermal dynamic changes across many fields, including studies of microorganisms (Kabanova et al., 2012) and pharmacological analysis. Essentially, useful bioactivity information can be obtained by measuring the thermo-kinetics of the drug-microorganism complex. *P. aeruginosa* was chosen as a model microorganism in this study because it is widespread in nature and a common bacteria found in wound infections (Wu et al., 2015). Furthermore, *P. aeruginosa* exhibited large thermo-kinetic changes, which improved testing and analysis.

Ultra Performance Liquid Chromatography (UPLC) is a highly efficient and accurate technique and is widely used to quantify the components of herbal drugs and generate chemical fingerprints. Chromatographic fingerprint methods can be used to characterize the holistic chemical profiles of herbal medicine. Chemical fingerprint is a useful tool for evaluation of herbal drugs' quality, differentiation of origin, identification of authenticity, and so on (Deng and Yang, 2013). Especially, it plays an important role in controlling the quality of herbal medicine without a reasonable strategy for ensuring the safety and efficacy of this herbal medicine (Zhang et al., 2014).

In the present study, microcalorimetry, UPLC, PCA, hierarchical cluster analysis (HCA), and canonical correlation analysis (CCA) were used in combination in order to distinguish the differences in bioactivity and chemical properties between LJF and LF, with the ultimate goal of characterizing their spectrum-effect relationships.

## Materials and methods

### Chemicals, reagents, and materials

Sixteen batches of Lonicera flowers buds were collected from various sources in China, as shown in Supplementary Table 1. Each species was authenticated by Professor Chunsheng Liu (School of Chinese Pharmacy, Beijing University of Chinese Medicine, Beijing, China).

Chlorogenic acid, 5-caffeoylquinic acid, 4-Dicaffeoylquinic Acid, 3,5-Dicaffeoylquinic acid, 3,4-Dicaffeoylquinic acid, and Luteoloside standards were purchased from Chengdu Pufei De biotech Co. Ltd. The purity of all compounds was higher than 98%. Acetonitrile, methanol were from Fisher Chemicals (Pittsburg, PA, USA). Formic acid was purchased from Fluka (Sigma-Aldrich, Gemrmany).

### Sample preparation

Fifty grams of fine powder from each batch of flower buds was weighed and then added to 500 ml of 70% ethanol solution. It was then extracted in a 100 Hz ultrasonic bath for 60 min, the solution was filtered, and the residue was extracted using the methods described above. After two rounds of extraction, the filtrate was combined and concentrated to dryness by evaporation. The residue was further dried in a vacuum freeze drier and then ground into powder.

### Instruments

UPLC fingerprints were measured using a Waters Acquity UPLC™ system (Waters, Milford, MA, USA) equipped with a binary solvent delivery pump, auto-sampler manager, column compartment, and a Photo-Diode Array (PDA) detector, connected to Waters Empower 2 software. The real-time metabolic HFP-time curves of *P. aeruginosa* were detected using a TAM 3114/3236 Bio-activity monitor (TA, Sweden) at 37°C connected to Picolog software. The baseline stability of the microcalorimeter was 0.2 mW for 24 h.

### Bacterial strain and recovery

*P. aeruginosa* (ATCC 27853) was supplied by the Clinical Examination Center of 302 Hospital of People's Liberation Army (PLA), Beijing, China and was stored in glycerinum solution at −80°C. Prior to experiments the stock solution was inoculated into 25 mL Luria-Bertani (LB) culture medium. It was then placed in a 37°C incubator shaker for 4 h with a rotational speed of 110 rpm and finally stored in a refrigerator at 4°C.

### Microcalorimetric measurements

All experimental manipulations were performed under sterile conditions. An ampoule method was used for microcalorimetry experiments and the ampoules were cleaned and autoclaved before use. The suspension of *P. aeruginosa* was added into equal volumes of culture medium containing different sample solutions of extract powder dissolved in medium. Each 20 mL ampoule contained 10 mL LB culture medium that included sample and bacteria. These were hermetically incubated at 37°C. The concentration of Lonicera flowers bud extract was 10 mg/mL. When the temperature of the sealed ampoules reached 37°C (this took about 30 min) a stable baseline was recorded, and the bacterial thermogenic curves were recorded until the output returned to the initial baseline.

### UPLC conditions

Samples were separated on an ACQUITY UPLC HSS T3 C18 column (2.1 × 100 mm, 1.8 μm) at 28°C using acetonitrile (A) and a 0.3% solution of formic acid in water (B). The gradient elution program utilized began with 10% A at 0–8.5 min, then 10–25% B at 8.5–9.5 min, 25% B at 9.5–16 min, 25–100% B at 16–25 min, and then maintained for 5 min. The flow-rate was kept constant at 0.2 mL/min. The effluent was monitored at 330 nm and the sample injection volume was 1.0 μL.

### Hierarchical clustering analysis

HCA is a statistic method used to determine the natural structure of objects in multidimensional profiles. This analysis can divide individuals or objects into specific clusters in a way that maximizes homogeneity within each cluster while also maximizing heterogeneity between clusters. In this study HCA was used to assess correlations in the UPLC fingerprints.

### Principal components analysis

Principal components analysis (PCA) is a statistical technique used to simplify a complex issue by transforming multiple variables into a small number of integrated variables while losing very little information. These integrated variables are known as principal components, and are uncorrelated linear combinations of the original variables, making these principal components superior compared to the original variables for many applications. In our study, PCA was used to process the thermo-kinetics parameters obtained from the power-time curve of the *P. aeruginosa* growth with different solutions of sample extracts.

### Canonical correlation analysis

CCA is a multivariate analysis used to study the correlation between two sets of variables. It is used for the dimension reduction of PCA and to extract the main principal components, and then describes the whole linear relationship of two sets of variables by the relevance of two principal components. In our study, CCA was used to analyze the relevance between the peak area values from the UPLC fingerprints and thermo-kinetic data from microcalorimetry.

## Result

### UPLC fingerprints

**Figure 1:**
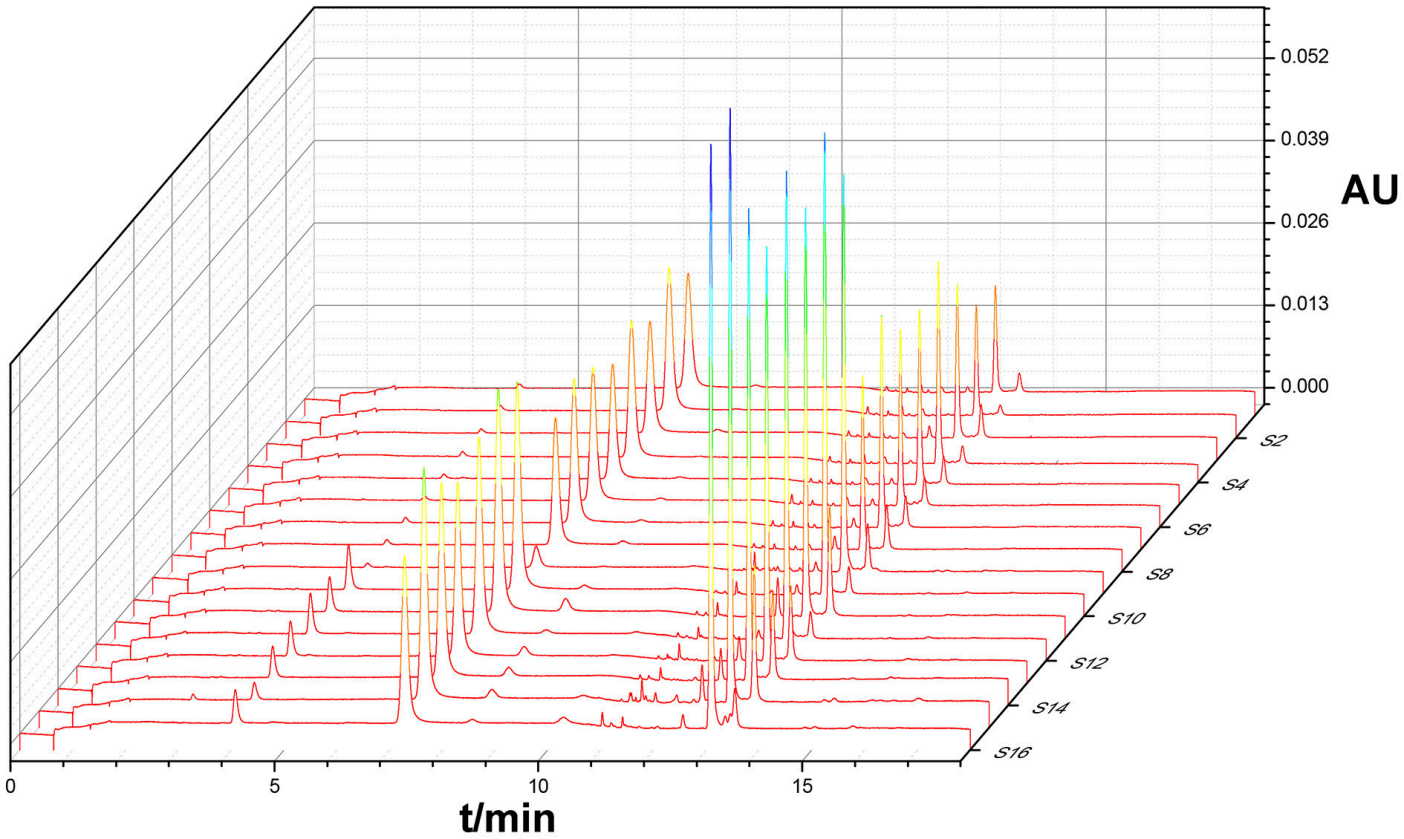
**UPLC fingerprints recorded for extracts from several batches**.

**Figure 2:**
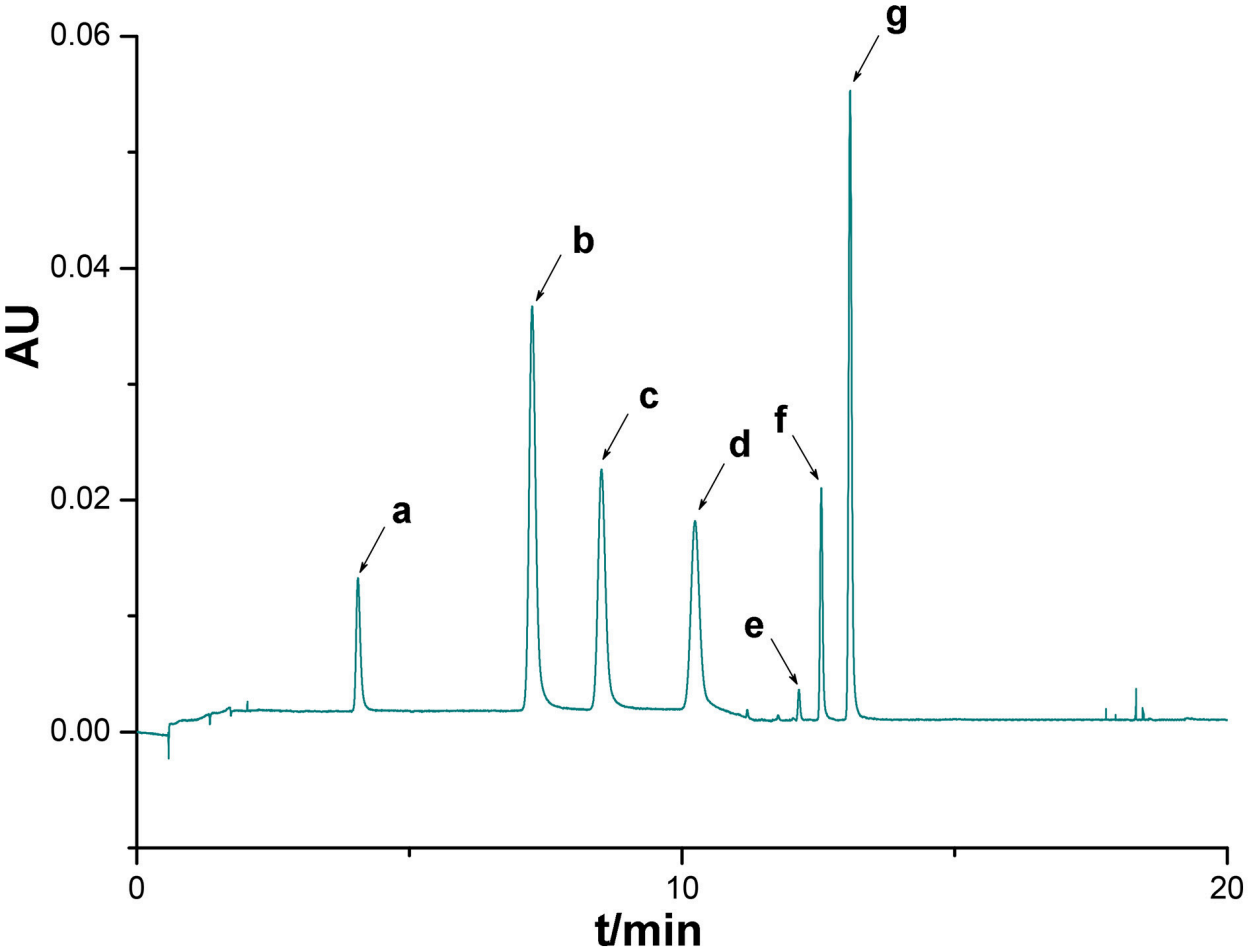
**UPLC chromatogram of a mixture of reference substances**. Seven peaks were identified by comparison with standard substances: 5-Caffeoylquinic acid (a), Chlorogenic acid (b), 4-Dicaffeoylquinic acid (c), caffeic acid (d), Luteoloside (e), 3,4-Dicaffeoylquinic acid (f), 3,5-Dicaffeoylquinic acid (g).

Under optimized conditions chromatograms were generated for all batches of bud extracts (Figure 1) as well as for a mixture of reference substances (Figure 2). There were similar chemical profiles across different bud sources, and seven common peaks were found by comparing ultraviolet spectra and UPLC retention times for the 16 chromatograms. The methods used to identify common peaks have been referenced in similar reports (Zheng et al., 2014).

Seven common characteristic peaks with large areas and good resolution from consecutive peaks were collected systematically from the 16 chromatograms. Because the S15 chromatogram lacked peak X7, this peak area was defined as 0. By comparing the chromatograms of the samples to that of the mixture of reference substances, peaks X1, X2, X4, X5, and X6 were identified as 5-caffeoylquinic acid, Chlorogenic acid, Luteoloside, 3,4-Dicaffeoylquinic acid, and 3,5-Dicaffeoylquinic acid, respectively. Peaks X3 and X7 remain unknown compounds. Some differences were noted among data collected for the common peaks (Table 1), such that the coefficients of variance (C.V.%) for all common peaks were larger than 31.71%. This is due to the diversity in the content of chemical compounds contained in different batches. As a next step, HCA was used to further elucidate the relative contents of the samples.

**Table 1:** **Peak areas of seven common peaks identified in UPLC results from bud samples**.

**Sample**	**Peak area of each peak**						
	**X1**	**X2**	**X3**	**X4**	**X5**	**X6**	**X7**
S1	3664	162885	2244	3660	3264	66799	16804
S2	4490	203475	3120	5121	2571	69998	11099
S3	3427	158112	2336	3754	6490	96113	26751
S4	4164	192949	3252	3975	2142	125294	16492
S5	4009	160814	2269	5318	3205	108962	22404
S6	3630	184491	2580	6579	3737	109596	23025
S7	4817	200251	3419	4522	5102	132187	27034
S8	4921	176323	2626	3697	7281	109592	32919
S9	3723	28679	7240	2593	6595	252109	39016
S10	37993	293813	5452	2623	5682	288757	21050
S11	32375	315170	7312	2947	20038	256660	79796
S12	37090	276626	6024	3598	4782	292943	21051
S13	30362	241644	5792	3016	14465	258679	54954
S14	26851	272180	5940	2502	17108	296894	66312
S15	14975	322970	12096	4812	22196	370947	0
S16	30004	233412	6733	2197	8978	363166	38768
C.V.%	91.65%	34.73%	55.01%	31.71%	77.37%	53.46%	66.79%

C.V.% = σ/μ*100; σ was the standard deviation, μ was the average value of peak area.

### HCA

**Figure 3:**
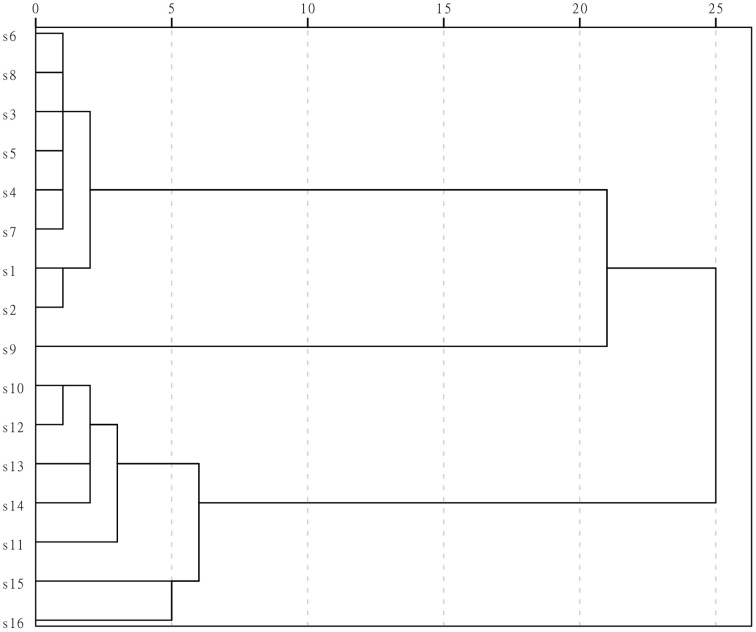
**Dendrogram of the HCA results for the UPLC fingerprints of 16 batches of extracts**. SPSS statistics software (SPSS for Windows 13.0, SPSS Inc., USA) was used to conduct the HCA and used the average linkage method and squared Euclidean distance.

Results of HCA showed that the fingerprints separated into three clusters, as shown in Figure 3: cluster one included S1–S8, cluster 2 contained only S9, and cluster three contained S10–S16. The samples separated into cluster one were batches of LJF from different places of origin, samples in cluster three were LF, and S9 in cluster two was the LF from Guizhou province. S9 is the species of LF closest to LJF in chemical profile, and is from the plant *Lonicera fulvotomentosa* Hsu et S. C. Cheng, one of the various species in the LF samples. The results of HCA showed that the extracts of LJF and LF from different origins had similar chemical fingerprints, yet HCA could initially separate the LJF and LF samples at the chemical level. Based on these results, a combination of HCA and UPLC with a PDA detector could, at least roughly, separate the bud extracts from two herbal drugs.

### HFP–time curves of *P. aeruginosa*

**Figure 4:**
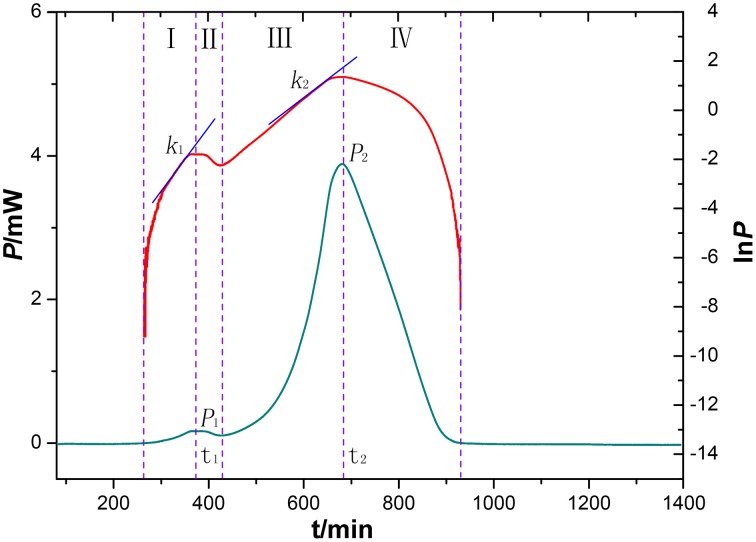
**HFP-time curves of ***P. aeruginosa*** growth without any extracts (blue), and the corresponding InP-time curve (red)**. The curves were divided into four phases (I-IV). The two curves reflect characteristic growth of *P. aeruginosa* at 37°C.

An HFP-time curve was used to record the power change of the ampoules containing medium inoculated with *P. aeruginosa* at 37°C. Because the ampoules were airtight, two peaks were recorded in the HFP-time curve, one showing the aerobic metabolism of the bacteria and the other due to the anaerobic metabolism. The InP-t curve reflected the metabolic character of *P. aeruginosa*. As is well known, bacterial growth can be divided into four phases, i.e., the initial exponential phase, the lag phase, the second exponential phase, and the decline phase. These phases are displayed in the HFP-time plot shown in the Figure 4.

By analyzing the HFP-time curve of the growth of *P. aeruginosa*, eight parameters (*k*_1_, *k*_2_, *P*_*m*1_, P_*m*2_, T_*m*1_, T_*m*2_, Q_1_, and Q_2_) were extracted from the four phases. These could be used to quantify the effects of various bud extracts on *P. aeruginosa*, as shown in Supplementary Table 2. The values of *k*_1_ and *k*_2_ are the maximum growth rate constants of the two peaks, *P*_*m*1_, P_*m*2_, T_*m*1_, and T_*m*2_ are the maximum heat output and the appearance time, respectively, of the two peaks, and Q_1_ and Q_2_ are the total quantity of heat in the aerobic and anaerobic stages of *P. aeruginosa* metabolism. Among the eight parameters, *P*_*m*1_, P_*m*2_, T_*m*1_, T_*m*2_, Q_1_, and Q_2_ could be obtained directly from the HFP-time curve. However, *k*_1_ and *k*_2_ were calculated using the following equations (Xie et al., 1988):

$${\text{P}}_{t}={\text{P}}_{0}\exp(\text{kt})\;or{\text{InP}}_{t}={\text{InP}}_{0}+\text{kt}$$

where P_0_ is the Heatflow-power at time *t* = 0 and P_*t*_ is the power at time *t*.

**Figure 5:**
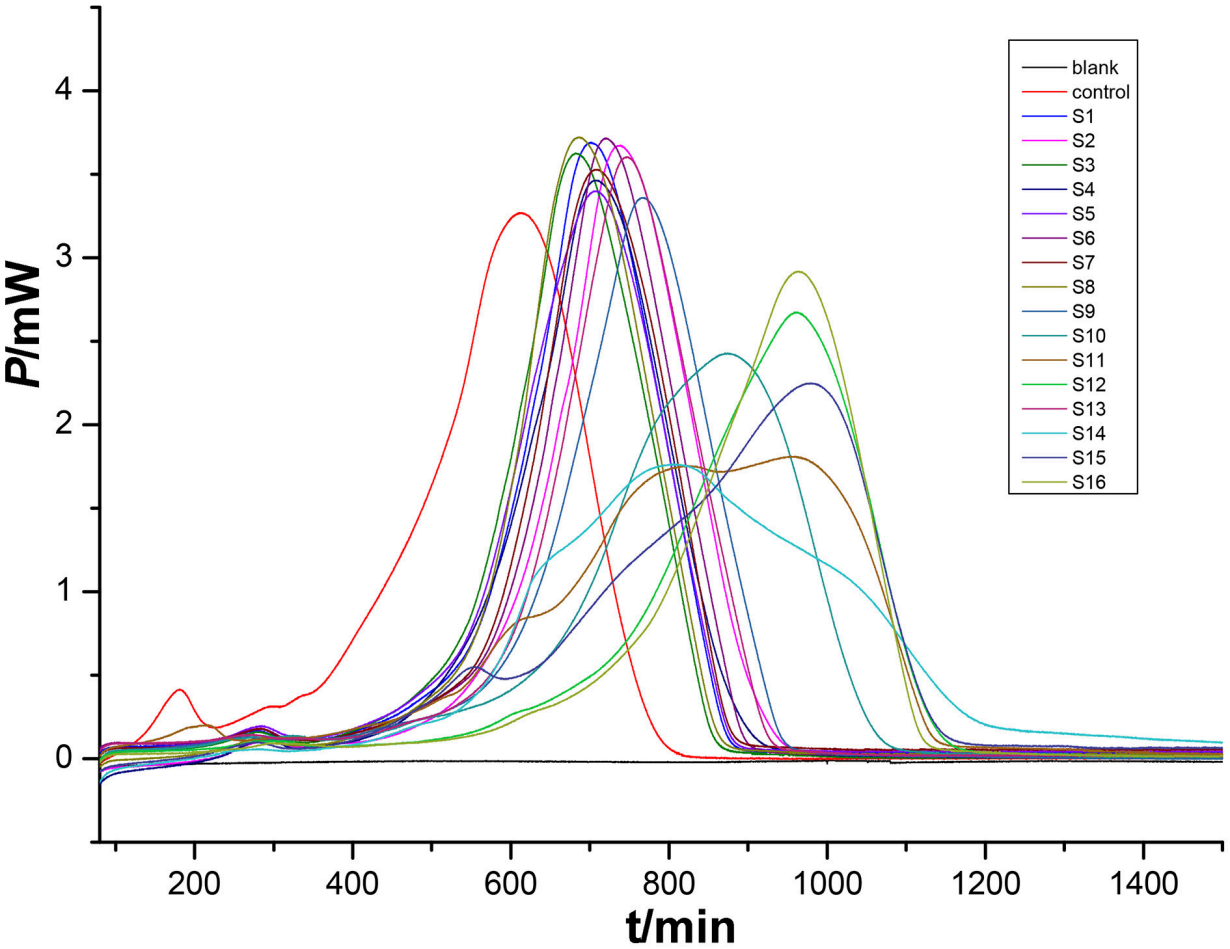
**HP-time curves of ***P. aeruginosa*** at 37°C in the presence of different batches of extracts**.

The HFP-time curves of *P. aeruginosa* after the addition of different bud extracts (Figure 5) were different from the control curve. This indicates that the bud extracts were able to influence bacterial growth to different extents. For example, the point of maximum heat of the *P. aeruginosa* growth was postponed for all samples, indicating that the medium that included extracts might inhibit the bacterial growth. This means that the bacterial culture would need more time to produce the defined number of bacteria needed to generate enough heat to achieve the intense signal. Furthermore, some curves measured using medium with extracts were severely deformed due to strong bioactivity on *P*. PCA was introduced as a further tool to analyze the thermokinetic parameters and further elucidate the extracts influence on bacteria.

### PCA

Through use of a PCA procedure it was possible to determine the most important variables, in other words, those variables that represent a majority of the total information. In this process, the values of thermo-kinetic parameters (*k*_1_, *k*_2_, T_*m*1_, T_*m*2_, *P*_*m*1_, P_*m*2_, Q_1_, and Q_2_) were analyzed using SPSS statistical analysis software. The results showed that the first three principal components accounted for about 80% of the information contained in the original eight parameters.

The relationships of the three principal components from eight indexes referencing the eigenvalue of the correlation matrix were expressed as the following equations:

$$\begin{array}{l} \text{Z}1=0.434\times k_{1}-0.525\times k_{2}+0.179\times {\text{T}}_{m1}-\text{0}.\text{740}\times {\text{T}}_{m2}\\ \;+0.554\times P_{m1}+0.940\times {\text{P}}_{m2}+0.595\times {\text{Q}}_{1}-0.802\times {\text{Q}}_{2}\\ \text{Z}2=0.536\times k_{1}+0.364\times k_{2}+0.822\times {\text{T}}_{m1}-\text{0}.\text{281}\times {\text{T}}_{m2}\\ \;-0.672\times P_{m1}+0.198\times {\text{P}}_{m2}-\text{0}.\text{668}\times {\text{Q}}_{1}-0.235\times {\text{Q}}_{2}\\ \text{Z}3=0.064\times k_{1}-0.733\times k_{2}+0.378\times {\text{T}}_{m1}+0.504\times {\text{T}}_{m2}\\ \;-0.053\times P_{m1}+0.010\times {\text{P}}_{m2}-0.062\times {\text{Q}}_{1}+0.062\times {\text{Q}}_{2} \end{array}$$

These result demonstrate that parameters *k*_2_, P_*m*2_, and T_*m*2_ might be the principle parameters, as they account for 79.37% of all components in the thermokinetic parameters (Supplementary Table 3).

**Figure 6:**
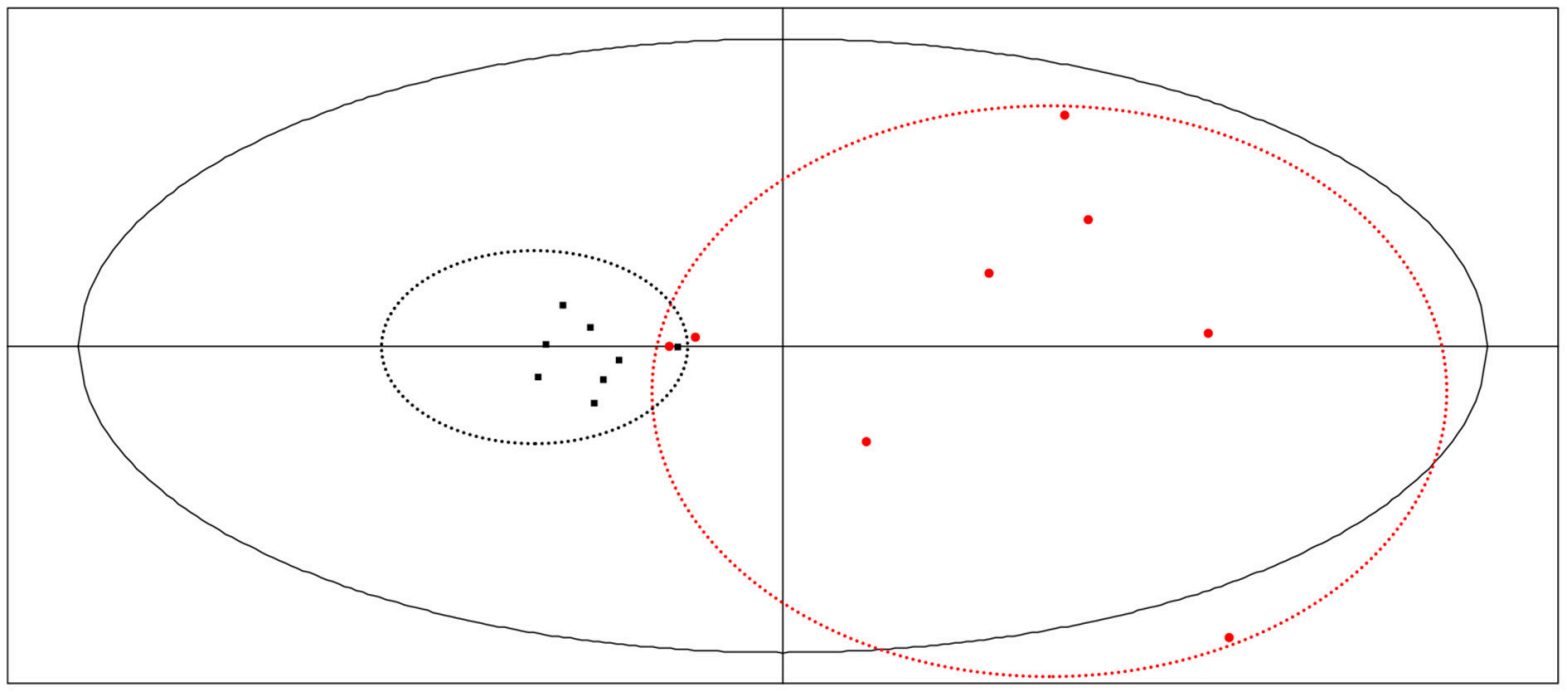
**PCA plot of microcalorimetry parameters generated using Simca-P software**. Samples of LJF appear as black points, while samples of LF appear as red points.

Analysis of the thermokinetic parameters in the PCA score plots generated by the Simca-P software (Figure 6) showed that the 16 batches of bud extracts were clearly divided into two groups, LF and LJF. These results demonstrated that there was obviously different effects on *P. aeruginosa* due to LJF vs. LF. From Figure 6, different batches of LJF showed little diversity, illustrating that the bioactivity of LJF was relatively consistent. In contrast, there was a large divergence among different batches of LF. S2 and S13 had similar bioactivity effects on *P. aeruginosa*, and S9 appeared to have effects similar to those of LJF. Overall, these results were similar to those from the HCA of UPLC data. In order to elucidate the relationships between thermokinetic data and UPLC fingerprints, CCA was introduced as a next step.

### Canonical correlation analysis

**Figure 7:**
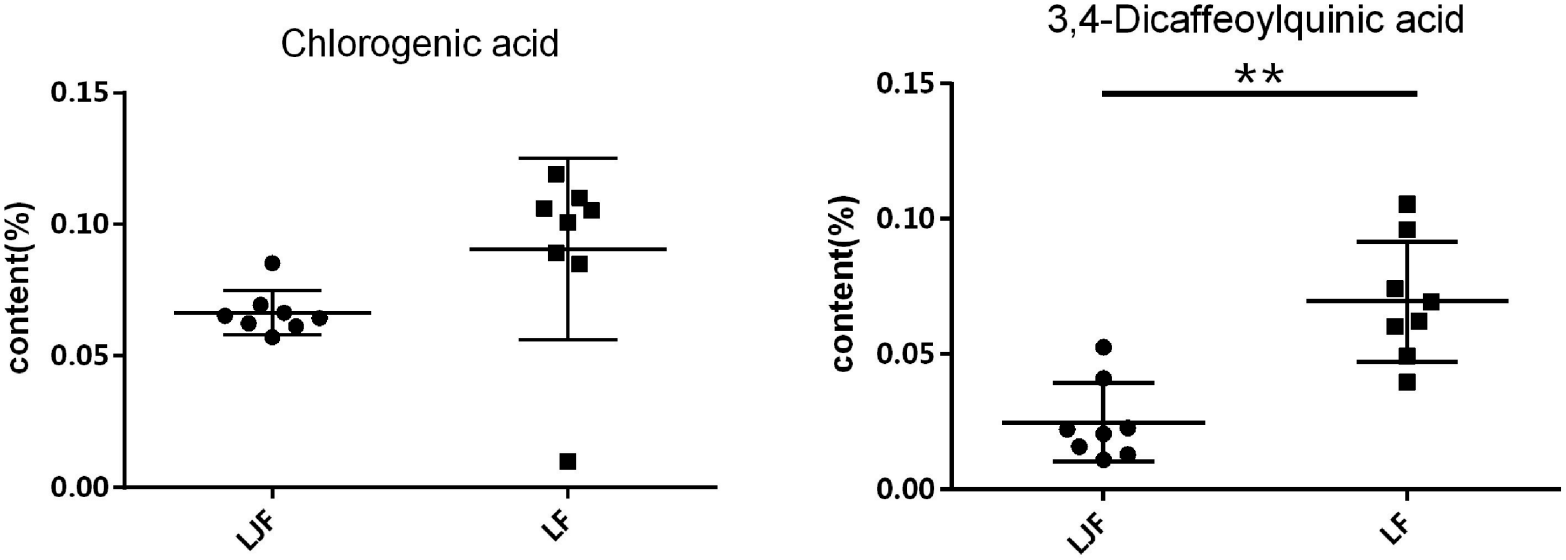
**The contents of Chlorogenic acid and 3,4-Dicaffeoylquinic acid in Lonicerae Japonicae Flos and Lonicerae Flos**. The points on the scatter plots represent the amounts of Chlorogenic acid or 3,4-Dicaffeoylquinic acid in batches of flower bud extracts. Error bars represent mean ± s.e.m. From a two-tailed unpaired Student's *t*-test, ^**^P < 0.01.

CCA was used to determine spectrum-effect relationships between area values of seven common peaks in the UPLC data and three principal components (*k*_2_, P_*m*2_, and T_*m*2_) extracted from the previously calculated thermodynamic parameters. The outcome of this analysis is shown in Table 2. Five peaks: X1, X2, X3, X5, and X6 were highly correlated (|*R*| > 0.6) with the major thermodynamic parameters. The correlation coefficients also showed that P_*m*2_ was negatively correlated with X1, X2, X3, X4, X6, and X7. This indicates that the thermokinetic parameter P_*m*2_ could be inhibited by these compounds. The X6 peak, previously identified as Isochlorogenic acid A was the most correlated (*R* = −0.7489) to biological effect. Other correlated peaks X1, X2, and X5 had been previously identified as neochlorogenic acid (5-caffeoylquinic acid), Chlorogenic acid, and 3,4-Dicaffeoylquinic acid. However, the X3 peak, which also showed good correlation, remains an unknown compound. Furthermore, parameter T_*m*2_ had a marked positive correlation (*R* > 0.6) with peaks X2 and X5, demonstrating that chlorogenic acid and 3,4-Dicaffeoylquinic acid, respectively, could delay the appearance of the second peak. Inhibition of P_*m*2_ and delaying T_*m*2_ are both antibacterial effects (Kong et al., 2011). Therefore, the above results indicated that chlorogenic acid and 3,4-Dicaffeoylquinic acid are the major antibacterial compounds in LJF and LF. Next the content levels of these compounds in LJF and LF were analyzed, as shown in Figure 7. The content of 3,4-Dicaffeoylquinic acid was significantly different between the two herbal drug samples, and could therefore be a marker for quality control in LJF and LF.

**Table 2:** **The correlation coefficients between the previously identified common characteristic peaks and previously calculated principal thermokinetic parameters**.

**Principal components**	**Peak No**.						
	**X1**	**X2**	**X3**	**X4**	**X5**	**X6**	**X7**
*K_2_*	0.1752	0.1726	0.0434	−0.2479	0.3128	0.1856	0.2523
P_*m*2_	−0.7091	−0.7153	−0.6935	0.4402	−0.7211	−0.7489	−0.6801
T_*m*2_	0.5158	0.7313	0.4998	−0.1612	0.6278	0.4213	0.5776

*Peak identifications: (X1) 5-Caffeoylquinic acid, (X2) Chlorogenic acid, (X3) unknown compound, (X4) Luteoloside, (X5) 3,4-Dicaffeoylquinic acid, (X6) 3,5-Dicaffeoylquinic acid, and (X7) unknown compound*.

## Discussion

Nowadays the strategy for quality control of herbal drugs depends mostly on measuring the content of constituents, which is correlated with the safety and efficacy of herbal medicine (Capasso et al., 2000). However, the relationships and efficacies of specific constituents in herbal drugs remain ambiguous. In this study, a bacterial model was used to evaluate the antibacterial effects of LJF and LF. The results indicated that the effects of LJF on *P. aeruginosa* were similar regardless of the geographical origins of LJF. This, suggested suggesting that the quality of LJF was more stable as compared to LF. Microcalorimetry could be used to provide further information regarding bioactivity (Zang et al., 2011). Furthermore, biological thermokinetic parameters can reflect antibacterial effects of whole compounds in the herbal extractions. Although chemical fingerprints could show the differences between most constituents in herbal medicine, it could not directly show the bioactivity of herbal drugs. Based on these observations, it is a promising strategy to combine the use of chemical fingerprints and microcalorimetry in order to ensure the quality of herbal medicine. In our study which utilized statistical analysis, two major components were found to be primarily responsible for the antibacterial effects of herbal drugs. It was particularly interesting that the content of one of these components, 3,5-Dicaffeoylquinic acid, was significantly different between LJF and LF. This could be used in the future as a new marker for quality control of LJF and LF.

Species and geographical origin influence the distinct content within plants (Canas et al., 2000). Only flower buds from *Lonicera japonica* Thunb. are defined as LJF. The LF samples were collected from three plant species for our study. The results of PCA showed that the samples of LJF were relatively concentrated although they were collected from different places. However, the LF samples were much more spread out. Most of the LF samples were from a single species, *Lonicera macranthoides* Hand.-Mazz., yet these samples were also quite discrete. This result demonstrates that when considering factors that can lead to different biological effects, geographical origin should obviously be included. After analyzing the geographical environment of the LJF and LF samples, it was determined that the sources of LJF were concentrated on the North China Plain, while most LF samples were widely distributed across mountainous Southwest China. Southwest China is quite different from the North China Plain in geographical characteristics, climate, and seasonality. As an adaptive consequence of the complicated ecology, the plants in the Southwest area conserve high levels of diversity, an effect which could contribute to the discrete bioactivities of the LF samples.

Ensuring the safety and efficacy of drugs involves multiple considerations and the quality of the drug must be fundamentally guaranteed. An important part of drug quality control is to ensure consistent medical and biological effects are delivered by the same drug dosage (Busse, 2000). In our research, there is a divergence in bioactivity between LJF and LF using the bacterial model. Separation of LF from LJF in the Chinese Pharmacopoeia could to a certain degree ensure the accuracy of clinical applications. However, microcalorimetry and chemical fingerprint results for *L. fulvotomentosa* Hsu et S. C. Cheng, a species of LF, closely approached those for LJF. This indicates that the buds of *L. fulvotomentosa* Hsu et S. C. Cheng may be used as LJF. Based on these complex results, there is an urgent need for further and more fundamental research on LJF and LF. There are many areas influenced by the modification of the Chinese Pharmacopoeia, such as history, economy, politics, civilization, and ethnic habits. Any change to the Chinese Pharmacopoeia could have far-reaching impacts. Therefore, the disaggregation of LJF and LF was not only a scientific question, but a much more comprehensive issue. The future will require that scientific researchers and administrators work together to study and develop relevant policies for promoting the development of the traditional Chinese medicine industry.

## Author contribution

ZS did the writing of paper and UPLC experiments as well as microcalorimetric experiments; ZL did the UPLC experiments; CL and YZ did the plant identification; MW and HS did the microcalorimetric experiments; XM did the statistical analysis; JW, YZ, and XX supervised the project. All the authors read and approved the final manuscript.

### Conflict of interest statement

The authors declare that the research was conducted in the absence of any commercial or financial relationships that could be construed as a potential conflict of interest.

## References

[B1] Busse W. (2000). The significance of quality for efficacy and safety of herbal medicinal products. Ther. Innov. Regul. Sci. 34, 15–23. 10.1177/009286150003400102

[B2] Canas S. Leandro M. C. Spranger M. I. Belchior A. P. (2000). Influence of botanical species and geographical origin on the content of low molecular weight phenolic compounds of woods used in Portuguese cooperage. Holzforschung 54, 255 10.1515/HF.2000.043

[B3] Capasso R. Izzo A. A. Pinto L. Bifulco T. Vitobello C. Mascolo N. (2000). Phytotherapy and quality of herbal medicines. Fitoterapia 71(Suppl. 1), S58–S65. 10.1016/S0367-326X(00)00173-8 10930714

[B4] Commission C. (2015). Pharmacopoeia of the People's Republic of China. Beijing: China Medical Science Press.

[B5] Deng J. Yang Y. (2013). Chemical fingerprint analysis for quality assessment and control of Bansha herbal tea using paper spray mass spectrometry. Anal. Chim. Acta 785, 82–90. 10.1016/j.aca.2013.04.056 23764447

[B6] Jiang P. Sheng Y. C. Chen Y. H. Ji L. L. Wang Z. (2014). Protection of Flos Lonicerae against acetaminophen-induced liver injury and its mechanism. Environ. Toxicol. Pharmacol. 38, 991–999. 10.1016/j.etap.2014.10.019 25461560

[B7] Kabanova N. Stulova I. Vilu R. (2012). Microcalorimetric study of the growth of bacterial colonies of *Lactococcus lactis* IL1403 in agar gels. Food Microbiol. 29, 67–79. 10.1016/j.fm.2011.08.018 22029920

[B8] Kong W. Wang J. Zang Q. Xing X. Zhao Y. Liu W.. (2011). Fingerprint-efficacy study of artificial *Calculus bovis* in quality control of Chinese materia medica. Food Chem. 127, 1342–1347. 10.1016/j.foodchem.2011.01.095 25214136

[B9] Li Y. Cai W. Weng X. Li Q. Wang Y. Chen Y.. (2015). Lonicerae Japonicae Flos and Lonicerae Flos: a systematic pharmacology review. Evid. Based Complement. Alternat. Med. 2015:905063. 10.1155/2015/905063 26257818 PMC4519546

[B10] Tae J. Han S. W. Yoo J. Y. Kim J. A. Kang O. H. Baek O. S.. (2003). Anti-inflammatory effect of *Lonicera japonica* in proteinase-activated receptor 2-mediated paw edema. Clin. Chim. Acta 330, 165–171. 10.1016/S0009-8981(03)00017-2 12636936

[B11] Wu M. Qu F. Zhao Y. Wang J. Su H. Chen C. (2015). Microcalorimetry and turbidimetry to investigate the anti-bacterial activities of five fractions from the leaves of *Dracontomelon dao* on *P. aeruginosa*. J. Thermal Anal. Calorimetry. [Epub ahead of print]. 10.1007/s10973-015-4932-2.

[B12] Xie C. Tang H. Song Z. Qu S. Liao Y. Liu H. (1988). Microcalorimetric study of bacterial growth. Thermochim. Acta 123, 33–41. 10.1016/0040-6031(88)80007-8

[B13] Zang Q. C. Wang J. B. Kong W. J. Jin C. Ma Z. J. Chen J.. (2011). Searching for the main anti-bacterial components in artificial *Calculus bovis* using UPLC and microcalorimetry coupled with multi-linear regression analysis. J. Sep. Sci. 34, 3330–3338. 10.1002/jssc.201100500 22058087

[B14] Zhang Z. Liang Y. Xie P. Chau F. Chan K. (2014). Chromatographic fingerprinting and chemometric techniques for quality control of herb medicines, in Data Analytics for Traditional Chinese Medicine Research, eds Poon J. Poon K. S. (Cham: Springer International Publishing). 10.1007/978-3-319-03801-8_8

[B15] Zheng Q. Zhao Y. Wang J. Liu T. Zhang B. Gong M.. (2014). Spectrum-effect relationships between UPLC fingerprints and bioactivities of crude secondary roots of *Aconitum carmichaelii* Debeaux (Fuzi) and its three processed products on mitochondrial growth coupled with canonical correlation analysis. J. Ethnopharmacol. 153, 615–623. 10.1016/j.jep.2014.03.011 24632114

