# Supplementary material for: Spectrum-Effect Relationships Between Chemical Fingerprints and Antibacterial Effects of Lonicerae Japonicae Flos and Lonicerae Flos Base on UPLC and Microcalorimetry
Source: Front Pharmacol. 2016 Feb 2;7:12. doi: 10.3389/fphar.2016.00012 (PMC4735347; doi:10.3389/fphar.2016.00012)
Supplement: Supplementary file 1 [file Table1.DOCX]

**Supplementary TABLE 1 Details of the sixteen flower bud samples**

| Sample number | source | Original plant | Official name in Chinese Pharmacopoeia |
| --- | --- | --- | --- |
| S1 | Julu, Xingtai, Hebei province(green buds) | *Lonicera japonica* Thunb | LONICERAE JAPONICAE LFOS |
| S2 | Julu, Xingtai, Hebei province(white buds) |  |  |
| S3 | Nangong, Xingtai, Hebei province(green buds) |  |  |
| S4 | Nangong, Xingtai, Hebei province(green buds) |  |  |
| S5 | Feixian, Linxi, Shandong province |  |  |
| S6 | Pingyi, Linxi, Shandong province |  |  |
| S7 | Xinmi, Zhengzhou, Henan province |  |  |
| S8 | Fengqiu, Xinxiang, Henan province |  |  |
| S9 | Anlong, Guizhou province( green buds) | *Lonicera fulvotomentosa* Hsu et S. C. Cheng | LONICERAE LFOS |
| S10 | Anlong, Guizhou province( white buds) | *Lonicera macranthoides* Hand.-Mazz |  |
| S11 | Xiaoshajiang, Longhui, Hunan province |  |  |
| S12 | Longjiawan village, Longhui, Hunan province |  |  |
| S13 | Longhui, Hunan province( green buds) |  |  |
| S14 | Xiushan, Chongqing city |  |  |
| S15 | Neijiang, Sichuan province(green buds) | *Lonicera hypoglauca* Miq |  |
| S16 | Neijiang, Sichuan province(white buds) |  |  |

**Supplementary TABLE 2 Thermokinetic parameters**

|  | Thermokinetic parameters | | | | | | | |
| --- | --- | --- | --- | --- | --- | --- | --- | --- |
| Samples | k_1_ | k_2_ | T_m1_ | T_m2_ | P_m1_ | P_m2_ | Q_1_ | Q_2_ |
| control | 0.2073 | 0.0075 | 180.93 | 613.83 | 0.4161 | 3.2706 | 27.8 | 778.3 |
| S1 | 0.02338 | 0.00891 | 277.00 | 700.33 | 0.1685 | 3.6897 | 14.7 | 742.7 |
| S2 | 0.00964 | 0.01052 | 293.5 | 738.50 | 0.1137 | 3.6734 | 8.6 | 755.3 |
| S3 | 0.01339 | 0.01062 | 278.33 | 681.50 | 0.1612 | 3.6256 | 14.4 | 728.3 |
| S4 | 0.01393 | 0.00948 | 291.17 | 706.16 | 0.1205 | 3.4636 | 7.6 | 734.8 |
| S5 | 0.01186 | 0.01161 | 282.00 | 708.00 | 0.1957 | 3.3997 | 18.4 | 741.2 |
| S6 | 0.01289 | 0.01329 | 282.00 | 719.50 | 0.1805 | 3.7161 | 17.6 | 736.8 |
| S7 | 0.01438 | 0.00953 | 283.83 | 706.67 | 0.1821 | 3.5289 | 14.8 | 713.4 |
| S8 | 0.01282 | 0.00565 | 288.50 | 686.00 | 0.1277 | 3.7232 | 10.5 | 731.4 |
| S9 | 0.00946 | 0.00771 | 264.50 | 766.83 | 0.1317 | 3.3608 | 13.2 | 717.2 |
| S10 | 0.01269 | 0.00925 | 324.00 | 870.50 | 0.1391 | 2.4289 | 5.4 | 806.6 |
| S11 | 0.00292 | 0.00597 | 215.00 | 953.83 | 0.2035 | 1.8107 | 15.4 | 826.0 |
| S12 | 0.01235 | 0.0043 | 276.17 | 958.50 | 0.1075 | 2.6737 | 15.0 | 740.3 |
| S13 | 0.00618 | 0.0078 | 274.33 | 747.00 | 0.1451 | 3.6048 | 12.0 | 729.7 |
| S14 | 0.0114 | 0.1479 | 275.83 | 805.33 | 0.0597 | 1.7647 | 5.5 | 789.2 |
| S15 | 0.01271 | 0.0031 | 279.50 | 978.17 | 0.1001 | 2.2491 | 8.9 | 799.6 |
| S16 | 0.01151 | 0.00554 | 293.67 | 962.50 | 0.0936 | 2.9185 | 12.7 | 723.8 |

**Supplementary TABLE 3** **Total variance explanation of PCA**

| component | Initial eigenvalues | | | Extraction sums of squared loading | | |
| --- | --- | --- | --- | --- | --- | --- |
|  | total | % of variance | cumulative | Total | %of variance | cumulative% |
| 1 | 3.234 | 40.424 | 40.424 | 3.234 | 40.424 | 40.424 |
| 2 | 2.166 | 27.076 | 67.5 | 2.166 | 27.076 | 67.5 |
| 3 | 0.95 | 11.871 | 79.371 | 0.95 | 11.871 | 79.371 |
| 4 | 0.733 | 9.157 | 88.528 |  |  |  |
| 5 | 0.6 | 7.502 | 96.03 |  |  |  |
| 6 | 0.248 | 3.105 | 99.135 |  |  |  |
| 7 | 0.058 | 0.726 | 99.861 |  |  |  |
| 8 | 0.011 | 0.139 | 100 |  |  |  |
